# 3D render volume CT reconstruction of the bones and arteries of the hind digit of the dromedary camel (*Camelus dromedarius)*

**DOI:** 10.1186/s40850-022-00151-8

**Published:** 2022-08-31

**Authors:** Samir A. A. El-Gendy, Basma M. Kamal, Mohamed A. M. Alsafy

**Affiliations:** 1 Department of Anatomy and Embryology, Faculty of Veterinary Medicine, Alexandria University, Abees 10th, Alexandria, Egypt; 2 Department of Anatomy and Embryology, Faculty of Veterinary Medicine, University of Sadat City, Sadat City, Egypt

**Keywords:** *A. digitalis plantaris communis III*, Angiography, Anatomy, *Camelus dromedarius*, 3D computed tomography, Digit, Phalanges

## Abstract

**Background:**

The 3D computed tomography produces detailed images of the digit bones in addition to the angiograph render volume 3D of the CT shows the relation between the arteries, bones, and tissues of the digit. Therefore, the present study used those imaging techniques to provide a complete description of the digit bones and arteries’ origin, distribution, and course and their relations with surrounding structures in the Dromedary Camel. Which would serve as a guide for surgeons and students in distinguishing normal digit structures. The study used eight hind limbs of four adult camels of both sexes (two males and two females), aged 9–15 years (Mean ± SD, 11.80 ± 2.59 years). The samples were injected with latex with lead oxide were undergone 3D render volume CT (128-slice multi-detector CT scanning protocol) and angiography x-rays.

**Results:**

The blood vessels and correlated structures such as bones, tendons, and ligaments were examined using 3D CT in all directions, which was easier to view than angiography and dissected specimens. The arterial supply to the camel’s hind foot was the *A. digitalis plantaris communis III*. The angiography render volume 3D of CT explained the blood supply of the bones and joints of digital regions and showed a good visualization of the many digit arteries. The metatarsals, the phalanges, and the sesamoid bones were visualized. *A. plantaris medialis superficialis, A. digitalis plantaris communis III, A. digitalis plantaris communis II* and *IV, A. interdigitalis, rami articularis medialis* and *lateralis* to the fetlock joint, *ramus medialis* and *ramus lateralis* of the *A. digitalis plantaris communis III*, *A. digitalis plantaris propriae III et IV abaxialis, A. digitalis plantaris propriae III et IV axialis, Ramus phalangis axialis and abaxialis of the first phalanx, Ramus phalangis axialis and abaxialis of the second and third phalanges, and A. metatarsae plantaris III* were visualized. The study discovered new blood vessel sources in dromedary camels, such as the *ramus articularis* to the fetlock and the *ramus plantaris phalangis abaxialis proximalis and distalis* of the first phalanx.

**Conclusions:**

The digital circulation angiography investigates the circulatory pattern of the camel hind digit, which can assist clinicians in diagnosing digit region affections. 3D CT explained improved visualization of bones and arteries, including many small branches in relation to surrounding structures, in some views better than others.

## Introduction

*Camelus dromedarius* features a singular foot structure than other animals [1]. It walks on large and wide pads for an extended time that bears its heavyweight within the slippery sand desert. Many studies have been conducted on the bones, joints, and ligaments of the foot of the dromedary camel [2–7].

Camel races and beauty shows were common in the Gulf region and Egypt, exploring the economic values of the Dromedary Camel. The frequent occurrence of lameness in racing camels is a big problem, therefore the understanding of the anatomical structure, biomechanics adjustment and custom [8, 9]. Lameness was more frequently observed in the hind limbs (46.6%) in most cases of lameness in 450 examined camels and the foot disorders were the most common disorders causing the lameness (59.05%) [10].

Most arteries and a few small arterial branches of the limb in the Dromedary Camel were studied by macroscopic anatomy and angiography [2, 11]. In Llama and Dromedary Camel, the caudal branch of the saphenous artery produces the plantar arteries and continues distally [12, 13].

The main arteries and some small branches of the foot of the forelimb and hind limb of the Dromedary and Bactrian camels were described by drawing photos from dissecting specimens [2, 14] respectively. The blood supply of the Dromedary Camel hind limbs and Llama feet were the plantar common digital III artery [12, 13]. While at the forelimb of the Bactrian Camel, it was derived from the palmar metacarpal artery III and palmar common digital artery III [14]. The blood supply of the axial part of the hind limb digits of the ox was derived from the dorsal metatarsal III artery to the dorsal surface of the foot and the plantar common digital III artery to the plantar surface of the foot. But, the abaxial part of the digit was supplied by the plantar common digital II and IV arteries [15]. The cattle forelimb-digits get the blood supply from the arteries: palmar metacarpal II, III, IV, the dorsal metacarpal III, and the palmar common digital II, III, and IV [16].

Angiography may be a technique for studying the structure, estimating the dimensions and diameter of vessels, wall integrity, and distribution. Moreover, it considers a diagnostic method for pathological conditions like ischemia, trauma, congenital malformation, and thrombosis [3].

Computed tomography scan and gross anatomy were performed by [17, 18] to describe and identify the morphological structures of the one-humped camel’s digit foot pad, metatarsus, and digits. Additionally, MRI was done by [19] to describe the forelimb’s foot structure. Angiographic techniques were performed to give a detailed pattern of distribution of the arterial blood supply in an adult dromedary camel’s foot, by [3, 20]. In order to investigate the fetlock joint in adult, healthy dromedary camels, [21] used computed tomography and magnetic resonance imaging, whereas [6, 7] used different types of CT and radiography to explain the structures of the fetlock and pastern joint in one-humped camels, respectively.

Computed tomographic angiography also generated the 3D reconstruction render volume data to reconstruct 3D models, especially when using volume-rendering (VR) techniques and MIPs [22, 23]. Three-dimensional reconstructions can define the anatomic relationship of the abdominal vessel [24]. CT angiography with 3D reconstruction provided accurate images of the kidney, renal pelvis, and ureter blood vessels [25].

The blood vessels provide an inherent contrast for CT imaging different from the bony structures. The administration of the contrast materials gets excellent visualization of the blood vessels in CT [26].

This study aimed to explain the bones and blood supply of the camel’s hind limb by angiography x-ray and CT that work out the origins, courses, situations, and arrangements of its branches regarding its surrounding structures by rendering volume 3D reconstruction on 360 degrees. The study provided a basis for further research on the anatomy of the camel by 3D reconstruction of CT bones and arteries of the digits.

## Material and methods

### Animals and specimen collection

The study used eight hind limbs of four adult camels of both sexes (2 males and 2 females), aged 9–15 years (Mean ± SD, 11.80 ± 2.59 years), and the age of the camels was determined by dentation according to [27]. And the bones of four camel digits from our anatomy laboratory for additional bone measurement. The camels had no clinical signs of orthopedic problems. They were examined clinically by palpation of metacarpal bones and joints for lameness prior to slaughter. The metatarsus with the digits were then separated from the hind limbs just under the tarsal joint, cooled, and imaged within two hours to minimize the post-mortem changes.

The collected specimens were kept in normal saline solution at ambient air for 2 hours. The *A. plantaris medialis superficialis* was canulated by 50 ml syringe at the apex of its origin and then thoroughly washed by warm normal saline solution NaCl 0.9% (Al Mottahedoon Pharma©) containing heparin calcium 5000 I. U (Cal-heparin Amoun©) to remove the remaining blood and clots in the arteries.

This study was approved by the local Animal Welfare and Ethics Committee, Faculty of Veterinary Medicine, Alexandria University, Egypt.

The nomenclature was adapted to [28].

### Injection of the arteries by contrast media

The arteries were first injected with normal saline and heparin then with a contrast radio-opaque mixture of 75 g red lead oxide powder (Jotun©) and 150 ml gum milk latex (Krember pigment, UK), which was diluted by ammonia until it was liquid to facilitate the junction process. The injection process occurred by a fixed 18-gauge plastic cannula through the *A. plantaris medialis superficialis* rapidly 2 to 3 minutes after preparation of mixture to overleap of the fast hardness of the contrast media.

### Angiography (radiopaque images)

Three prepared samples were imaged in three standard radiographic views; dorsoplantar, mediolateral, and oblique planes. The images were captured on a phosphor plate using Toshiba 500 MA fixed X-ray machine with an output of 80 Kv and 20 mA. The time was adjusted automatically with the mA, and the images were digitally processed using a CR reader (R30-X. Agfa, Japan).

### Computed tomography (3D render volume CT) (128-slice multi-detector CT scanning protocol)

For the scanning process of the camel hind foot, two limbs without contrast media and three samples with contrast media were placed on its plantar surface on the table, and secured in foam material to avoid any movement that may distress scanning.

The CT angiography examinations were performed on a MDCT scanner (Multidetector computed tomography) with 128 detectors (Aquilion; Toshiba Medical Systems, Tokyo, Japan) with a rotation time of 300 msec, and slice collimation of 128 × 0.6 mm using a continuous helical scan mindose technique. After obtaining a scout view to determine the 3D-CTA scan range, image acquisition began 6 seconds after the signal density level reached the predefined threshold of 100 Hounsfield units (HU). Parameter value distance source-isocenter 170 mm, distance isocenter-detector 39 mm. The number of pixels per detector row 128 detector rows, nominal tube voltage range 30–65 kV and 200 mA added filtration 0.5 mm, al collimation 46.5 mm, scintillator material CsI, scintillator thickness 0.6 mm [29].

The *A. plantaris medialis superficialis*-filled with contrast media was imaged to acquire data of the first blood-vessel generations, being a similar number as obtained by the angiography technique. The CT images were reconstructed with the optimal reconstruction parameter for blood vessels, bone, and ligaments. At first, a set of cross-sectional tomography slice by the slice was reconstructed. In the end, all images were stacked together sequentially to obtain a 3D image model of the object being inspected. The reconstruction algorithm was done with octopus software and converted to digital imaging and communications in medicine (DICOM) format [30].

### Bone morphometry

The length and width of the shaft, proximal and distal ends of the phalanges were measured by the ImageJ program from the reconstructed 3D CT images and from the bones of four camel digits from our anatomy laboratory, and the mean and standard error was done by Microsoft excel.

### Gross morphology

The three digits that were injected with latex and lead oxide for angiography examination were dissected for macroscopic evaluation of the *A. plantaris medialis superficialis* and its branches.

## Results

3D render volume reconstruction of CT explained the details of digits bones. Angiography 3D reconstruction of CT showed the relation between arteries, bones, and ligaments in 3D form and gave many photos by 360 degrees that showed a good visualization of the many small branches and their directions at communication. The 3D CT produces detailed images of the bones, arteries, and tissues of the foot, so the present study used those imaging techniques to describe the foot bones and arteries’ origin, distribution, and course in dromedary camel. The course of the blood vessels and the correlated structures like bones and ligaments have been presented by the 3D CT in all directions, which was better in viewing than the angiography and dissected specimens, especially the superficial branches, small branches, and deep branches.

### The bony structure of the digit

**Fig. 1:**
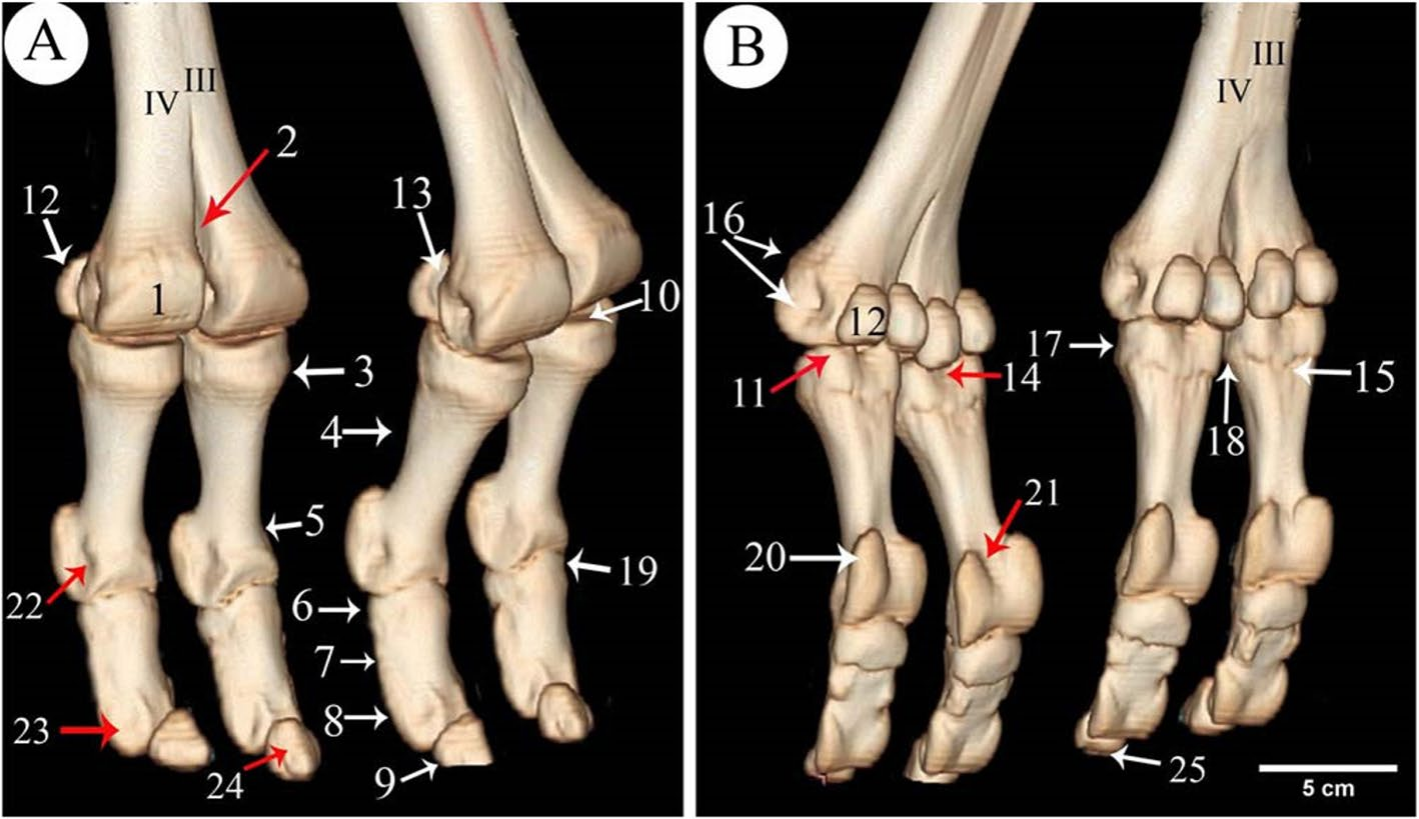
3D render volume reconstruction of the CT of the right and left hind digit bones of 11 years she-camel. **A** Dorsolateral view. **B** Plantarolateral view. 1. Trochlea of the metatarsal articular surface, 2. Intertrochlear notch, 3. The proximal extremity of the 1st phalanx, 4. The shaft of the 1st phalanx, 5. The distal extremity of the 1st phalanx, 6. The proximal extremity of the 2nd phalanx, 7. The shaft of the 2nd phalanx, 8. The distal extremity of the 2nd phalanx, 9. 3rd phalanx, 10. The proximal articular surface of the 1st phalanx, 11. Abaxial articular facet, 12. Proximal sesamoid bone, 13. The articular surface of the sesamoid with the distal extremity of the metatarsal bone, 14. Sagittal notch, 15. Rough triangular area, 16. Tubercle and depression on the abaxial aspect of the metatarsal distal extremity, 17. The rough area on the abaxial aspect of the 1st phalanx proximal extremity, 18. Transverse ridge & rough area on the axial aspect of the proximal extremity of the 1st phalanx, 19. An extensor process on the transverse ridge at the proximal extremity, 20. Large abaxial prominence, 21. Groove at the distal extremity of the 2nd phalanx, 22. Depression of the abaxial collateral ligament at the distal extremity of the 1st phalanx, 23. Depression for the ligaments on the abaxial of the distal extremity of the 2nd phalanx, 24. The parietal surface of the 3rd phalanx, 25. The solar surface of the 3rd phalanx, III. Third metatarsal bone, IV. Fourth metatarsal bone

CT oblique dorsal and plantar views of the digit regions went through 3D render volume reconstruction. That gave us a better visualization of the bones of the digits from all sides. Slight flexion of the fetlock joint gave us a chance to explore the articular surfaces of the distal extremity of the metatarsal, proximal extremities of the 1st phalanx and the articular surface of the sesamoid bones (Fig.1). The 3D reconstruction of the bone showed the bones of the lateral and medial sides of the same leg, and the bones of the right and left limbs were nearly similar in size. The measurements of the phalanges (Table 1) indicated that the left side was a slight increase in values over the right side and the lateral sides had higher values than the medial sides.

**Table 1:** The means of the morphometric values of the proximal, middle, and distal phalanges of the camel hind limb by mm

Parameters	Proximal phalanx				Middle phalanx				Distal phalanx			
	Right		Left		Right		Left		Right		Left	
	Medial	Lateral	Medial	Lateral	Medial	Lateral	Medial	Lateral	Medial	Lateral	Medial	Lateral
Length	99.8 ± 5.6	100.9 ± 6.9	99.5 ± 5.86	101.4 ± 5.99	61.5 ± 3.6	62.7 ± 4.2	61.8 ± 6.2	63.9 ± 5.8	21.3 ± 2.2	22.4 ± 1.4	21.5 ± 2	22.5 ± 1.7
Proximal width	38.1 ± 1.9	39.1 ± 1.8	39.2 ± 1.8	40.1 ± 1.3	29.7 ± 1.8	30 ± 1.3	30.1 ± 1.3	30.4 ± 2.1	19.8 ± 1.9	20.7 ± 2.3	21.1 ± 2.3	21.3 ± 2.6
Middle width	22.3 ± 1.9	21.4 ± 2.0	22.4 ± 2.6	23 ± 1.6	28,2 ± 2.3	28.1 ± 2.3	28 ± 2.5	28 ± 2.9	7.2 ± 1.3	7.4 ± 1.8	7.8 ± 2.1	8 ± 1.8
Distal width	37.1 ± 2.6	37.2 ± 2.3	38.3 ± 2.3	38.5 ± 2.9	37 ± 2.3	36 ± 2	37 ± 2.8	36 ± 2.6	3.3 ± 1.4	3.5 ± 1.9	4.1 ± 1.3	4.2 ± 2.6

The 1st phalanx was the longest bone of the digit bones. It was a nearly semi-cylindrical shape. Its proximal articular surface was a slightly concave compressed circle (Figs. 1/3,4; 2/25; 4/23; 7/1st; 8/13) and had two facets separated by an intermediated sagittal notch. These facets have articulated with the sesamoid bones (Fig. 1/12,14). This extremity articulates with the distal extremity of the 3rd & 4th metatarsal bones (Fig. 1/11,12), they form the fetlock joint (Fig. 4/30), rough areas on the sides of the proximal extremity, in addition to the transverse ridge on the axial side (Figs. 1/17; 4/35) The shaft had a roughly triangular area at the proximal third of the planter surface (Fig. 1/15). The distal articular surface of the 1st phalanx was strongly convex (Figs. 1/5; 4/29), and it had a proximal extension to the dorsal side and a greater extent to the plantar side, which was terminated by axial and abaxial prominences separated by a groove; the abaxial protrusion was larger than the axial one, and a depression on the sides of the distal extremities (Figs. 1/20,21,22; 4/36).

**Fig. 2:**
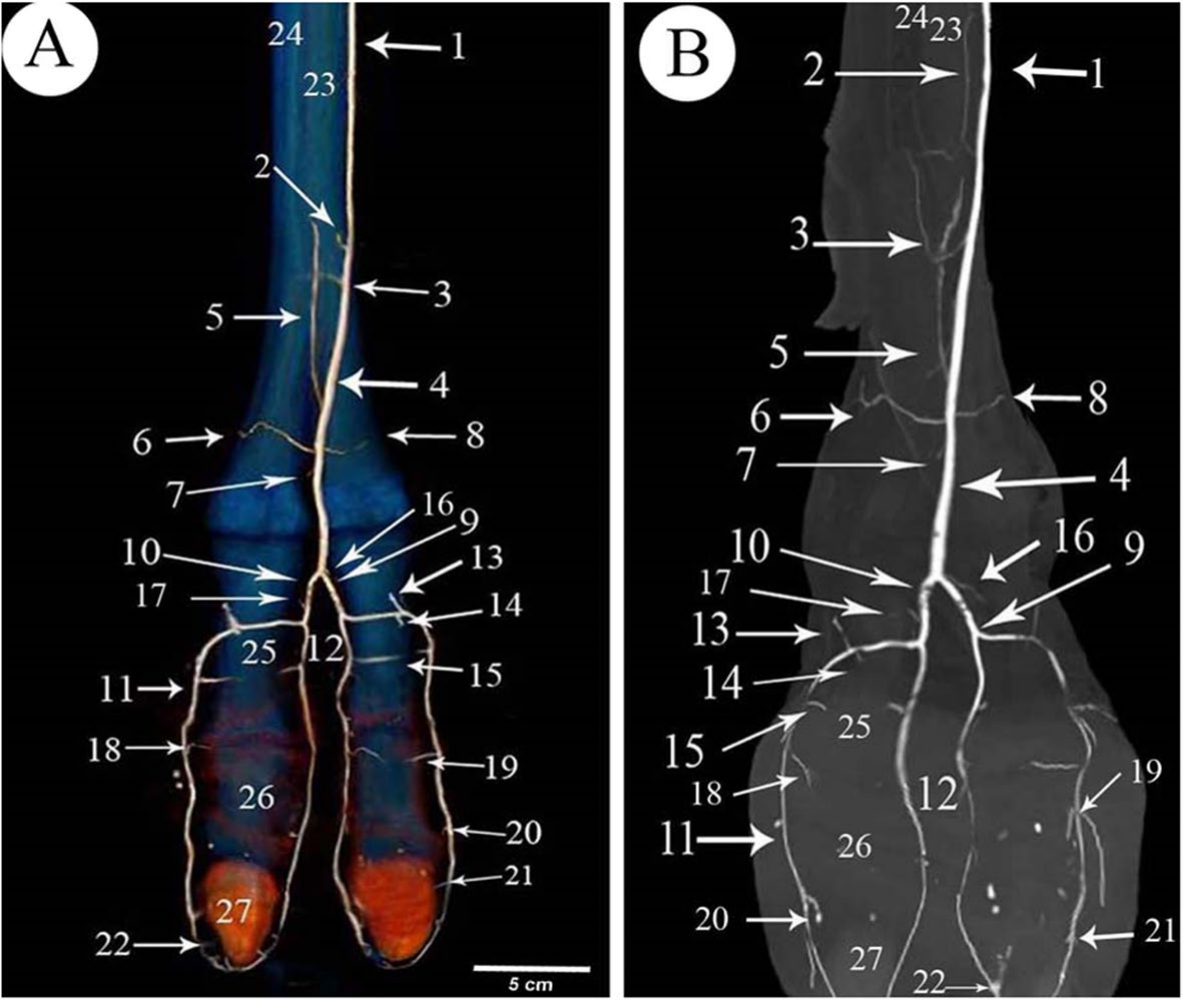
Plantar view of the metatarsus and phalanges of the left hind limb of 13 years male camel showed the *A. digitalis* plantaris communis III and its branches. **A** 3D render volume of CT image. **B** CT image. 1. *A. plantaris* medialis superficialis, 2. *A. digitalis* plantaris communis II, 3. *A. digitalis* plantaris communis IV, 4. *A. digitalis* plantaris communis III, 5. A. metatarsae plantaris III, 6. A. metatarsae plantaris IV, 7. Ramus interdigitalis, 8. A. metatarsae plantaris II, 9. *A. plantaris* medialis superficialis of the *A. digitalis* plantaris communis III, 10. Ramus lateralis of the *A. digitalis* plantaris communis III, 11. *A. digitalis* plantaris propriae III et IV abaxialis, 12. *A. digitalis* plantaris propriae III et IV axialis, 13. Ramus plantaris phalangis abaxialis proximalis, 14. Ramus plantaris phalangis abaxialis distalis of the first phalanx, 15. Ramus plantaris of the first phalanx, 16. Ramus articularis medialis to the fetlock joint from the *A. digitalis* plantaris communis III, 17. Ramus articularis lateralis to the fetlock joint from the Ramus lateralis of the *A. digitalis* plantaris communis III, 18. Ramus articularis of the pastern joint, 19. Ramus plantaris phalangis axialis and abaxialis of the second phalanx, 20. Ramus articularis of the coffin joint, 21. Ramus dorsalis phalangis axialis and abaxialis of the third phalanx, 22. Arcus terminalis, 23. Metatarsal bone III, 24. Metatarsal bone IV, 25. First phalanx, 26. Second phalanx, 27. Third phalanx

The 2nd phalanx was somewhat half of the 1st phalanx. And it was compressed dorsoplantarly, convex dorsally and flattened plantarly. Its proximal extremity was an elliptical shape with concave articular surface. An extensor process appeared on the transverse ridge of the proximal extremity of the 2nd phalanx (Figs. 1/6,7,8,19; 2/26; 4/24; 7/2nd; 8/14). Its distal extremity had depression on each side for ligaments attachment (Figs. 1/23; 4/37), and its distal articular surface was convex and extended on the dorsal and plantar surfaces.

The 3rd phalanx appeared wedge shape, its apex directed dorsally, and the articular surface somewhat flat (Figs. 1/9,24,25, 2/27; 4/25; 7/3rd; 8/15). The bones formed the fetlock, pastern, and coffin joints respectively (Fig. 4/31,32,33).

Features such as the trochlea, intertrochlear notch, tubercle and depressions on the axial and abaxial aspects were evident on the distal extremity of the 3rd and 4th metatarsal bones (Figs. 1/1, 2, 16; 4/34).

### *A. digitalis* plantaris communis III

**Fig. 3:**
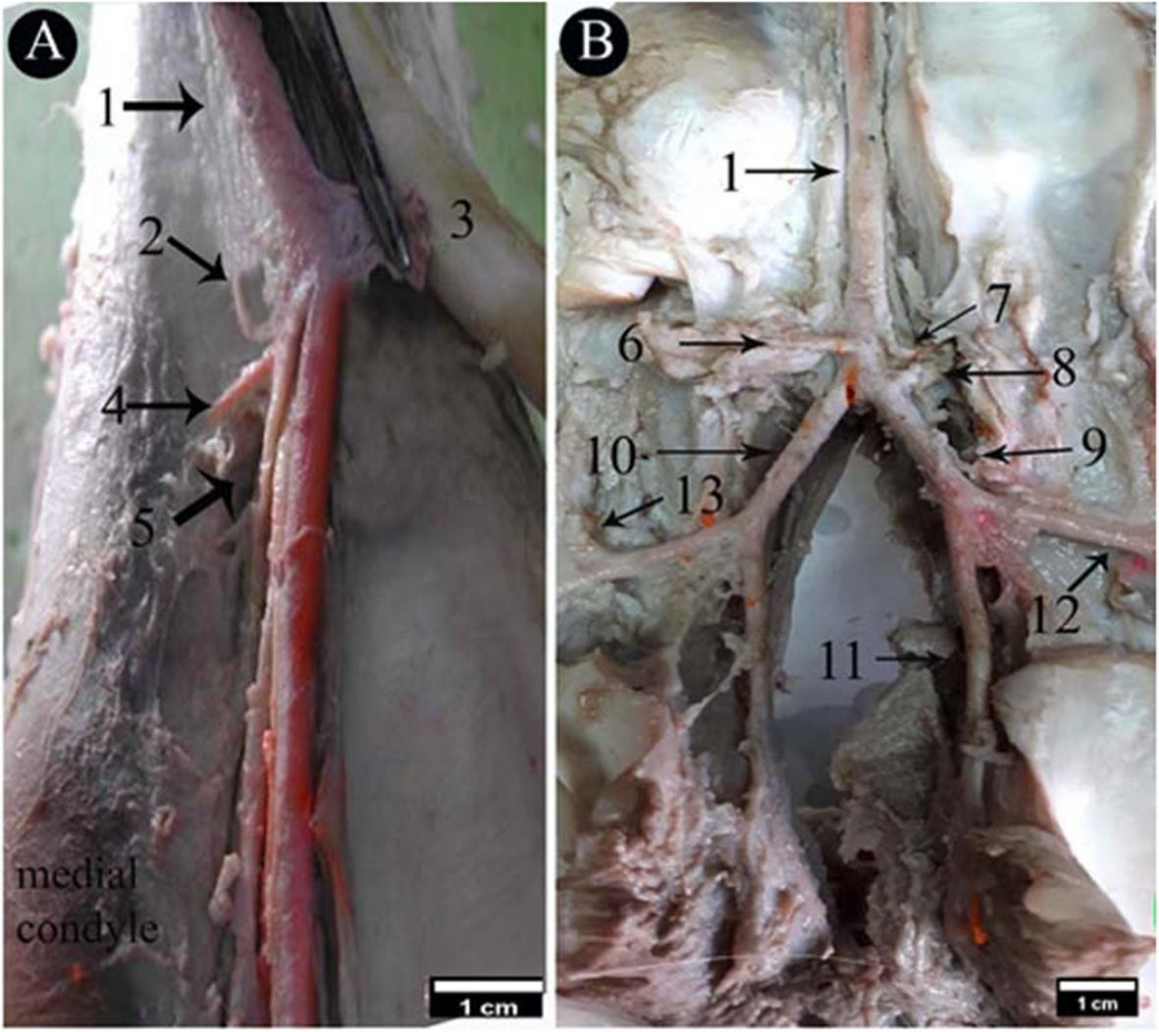
Plantar view of the dissected cadaver of the proximal and distal parts of the metatarsus (**A**), and phalanges (**B**) of the right hind limb of 9 years she-camel. 1. *A. digitalis* plantaris communis III, 2. A. metatarsae plantaris III, 3. Reflected deep digital flexor tendon, 4. A. metatarsae plantaris II, 5. Ramus interdigitalis, 6. Ramus articularis lateralis to the fetlock joint from the *A. digitalis* plantaris communis III, 7. Ramus articularis medialis to the fetlock joint from the *A. digitalis* plantaris communis III, 8. Ramus articularis to the fetlock joint from the ramus medialis of the *A. digitalis* plantaris communis III, 9. Ramus medialis of the *A. digitalis* plantaris communis III, 10. Ramus lateralis of the *A. digitalis* plantaris communis III, 11. *A. digitalis* plantaris propriae axialis, 12. *A. digitalis* plantaris propriae abaxialis, 13. Ramus plantaris phalangis abaxialis proximalis of the first phalanx

**Fig. 4:**
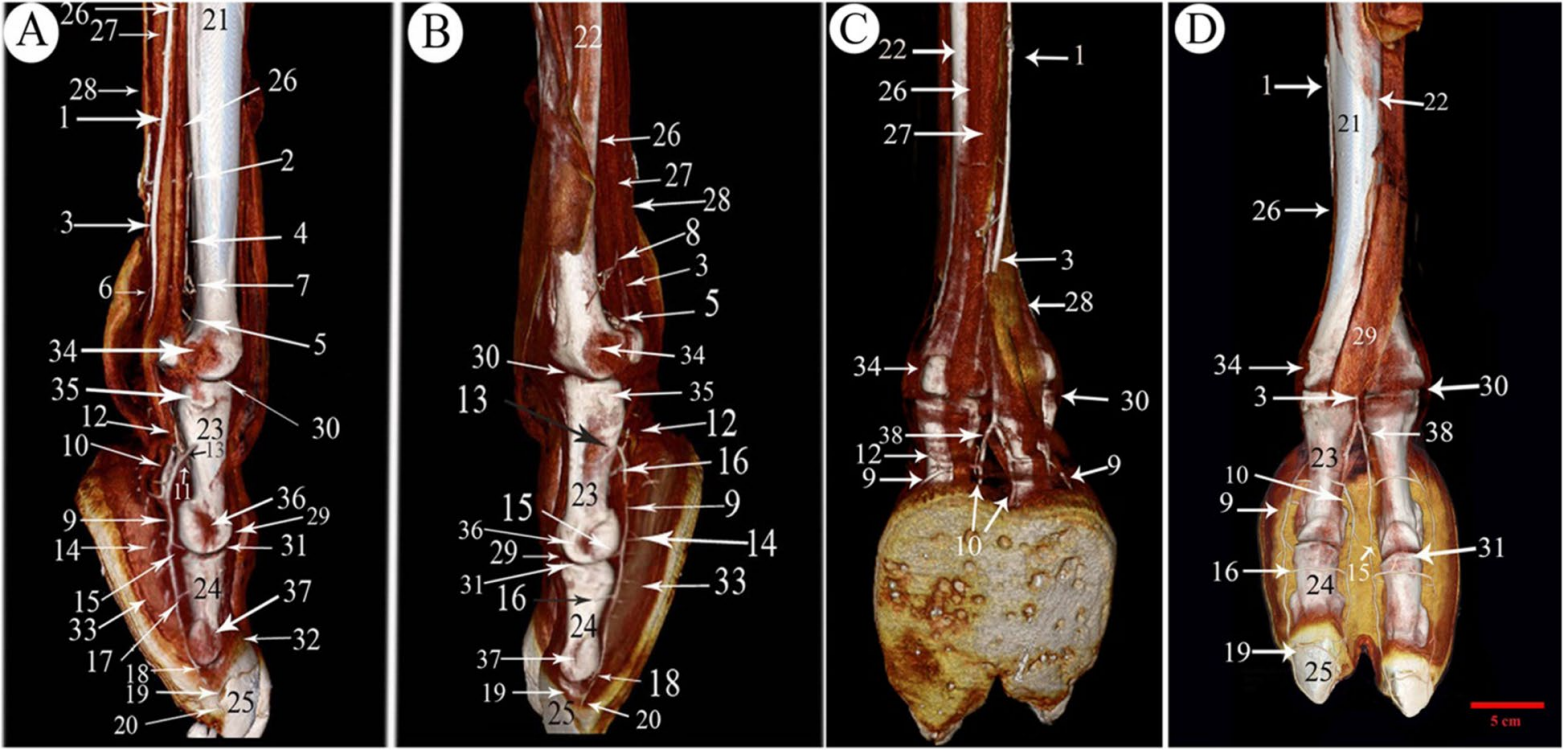
Medial (**A**), lateral (**B**), plantar (**C**) and dorsal (**D**) views of the 3D render volume of the CT at the metatarsal and digit regions of the left hind limb of 13 years male camel showing the *A. digitalis* plantaris communis III and its branches. 1. *A. plantaris* medialis superficialis, 2. *A. digitalis* plantaris communis II, 3. *A. digitalis* plantaris communis III, 4. A. metatarsae plantaris III, 5. Ramus interdigitalis, 6. Ramus superfecialis of *A. digitalis* plantaris communis III, 7. A. metatarsae plantaris II, 8. A. metatarsae plantaris IV, 9. *A. digitalis* plantaris propriae III et IV abaxialis, 10. *A. digitalis* plantaris propriae III et IV axialis, 11. Ramus plantaris phalangis abaxialis distalis of the first phalanx, 12. Ramus plantaris phalangis abaxialis proximalis of the first phalanx, 13. Ramus dorsalis phalangis abaxialis, 14. Ramus tori digitalis abaxialis, 15. Ramus articularis of the pastern joint, 16. Ramus plantaris of the first phalanx, 17. Ramus dorsalis phalangis abaxialis of the 2nd phalanx, 18. Ramus articularis of the coffin joint, 19. Ramus coronalis, 20. Arcus terminalis, 21. Third Metatarsal bone, 22. Fourth metatarsal bone, 23. First phalanx, 24. Second phalanx, 25. Third phalanx, 26. Middle interosseous muscle, 27. Deep digital flexor tendon, 28. Superficial digital flexor tendon, 29. Common digital flexor tendon, 30. Fetlock joint, 31. Pastern joint, 32. Coffin joint, 33. Digital cushion, 34. Depression on the abaxial aspect of the metatarsal distal extremity, 35. Transverse ridge & rough area on the axial aspect of the proximal extremity of the 1st phalanx, 36. Depression of the axial collateral ligament at the distal extremity of the 1st phalanx, 37. Depression for the ligaments on the axial of the distal extremity of the 2nd phalanx, 38. Ramus lateralis of the *A. digitalis* plantaris communis III

**Fig. 5:**
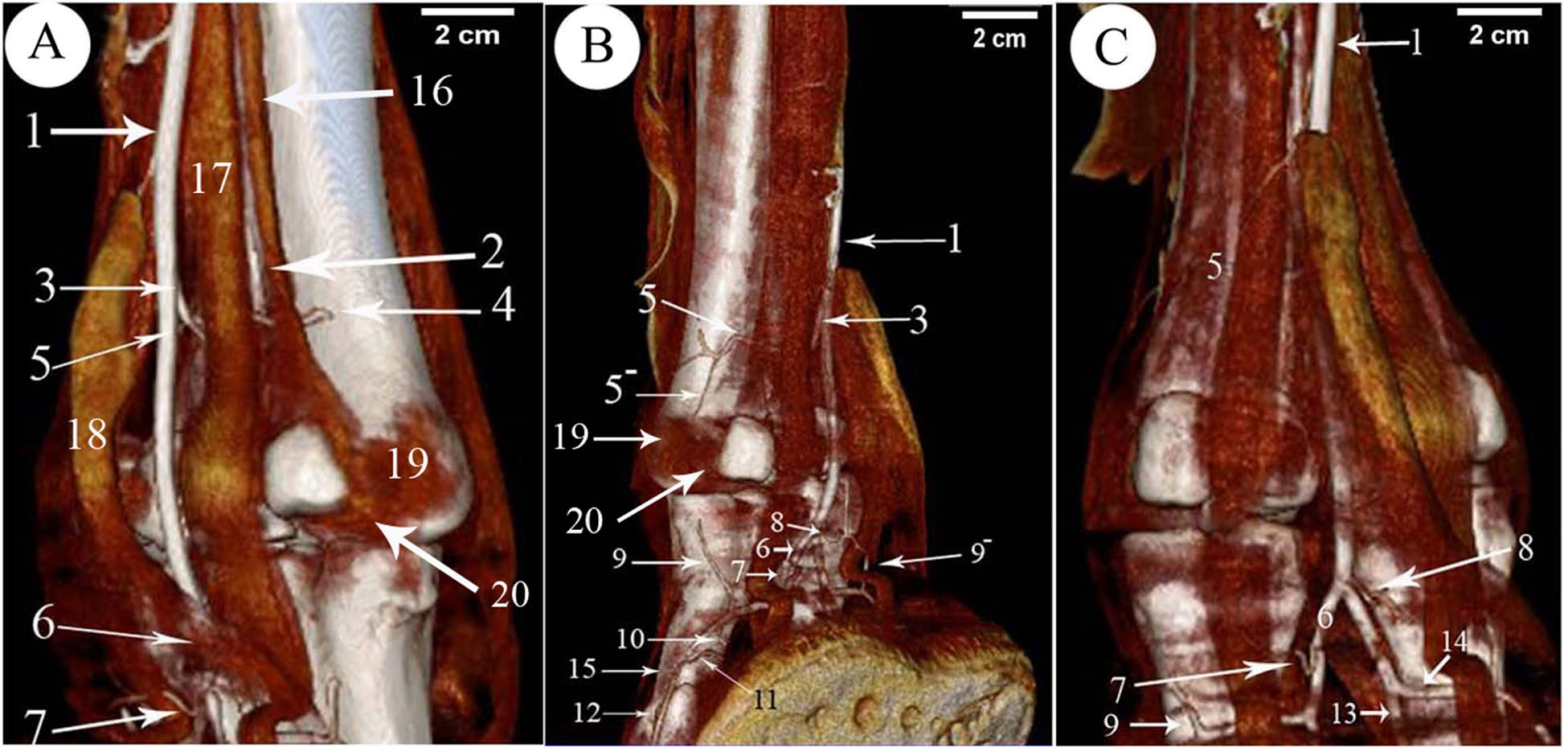
Plantomedial (**A**), plantarolateral (**B**), and plantar (**C**) views of the 3D render volume of the CT at the distal extremity of the metatarsal, fetlock and digit regions of the left hind limb of 13 years male camel showing the *A. digitalis* plantaris communis III and its branches. 1. *A. digitalis* plantaris communis III, 2. A. metatarsae plantaris III, 3. Ramus anastomoticus cum between 1&2, 4. A. metatarsae plantaris II, 5. A. metatarsae plantaris IV, 5^−^. Ramus articularis to the fetlock joint of the A. metatarsae plantaris III, 6. Ramus lateralis of the *A. digitalis* plantaris communis III, 7. Ramus articularis lateralis to the fetlock joint, 8. Ramus articularis medialis to the fetlock joint, 9. Ramus plantaris phalangis abaxialis proximalis of the first phalanx of the *A. digitalis* plantaris propriae IV abaxialis, 9^−^. Ramus plantaris phalangis abaxialis proximalis of the first phalanx of the *A. digitalis* plantaris propriae III abaxialis, 10. Ramus plantaris phalangis abaxialis distalis of the first phalanx, 11. Ramus plantaris of the first phalanx, 12. Rami articularis abaxialis of the pastern joint, 13. *A. digitalis* plantaris propriae III et IV axialis, 14. *A. digitalis* plantaris propriae III abaxialis, 15. *A. digitalis* plantaris propriae IV abaxialis, 16. Middle interosseous muscle, 17. Deep digital flexor tendon, 18. Superficial digital flexor tendon, 19. Axial & abaxial collateral ligament of the fetlock joint, 20.Axial & abaxial collateral sesamoidin ligament

**Fig. 6:**
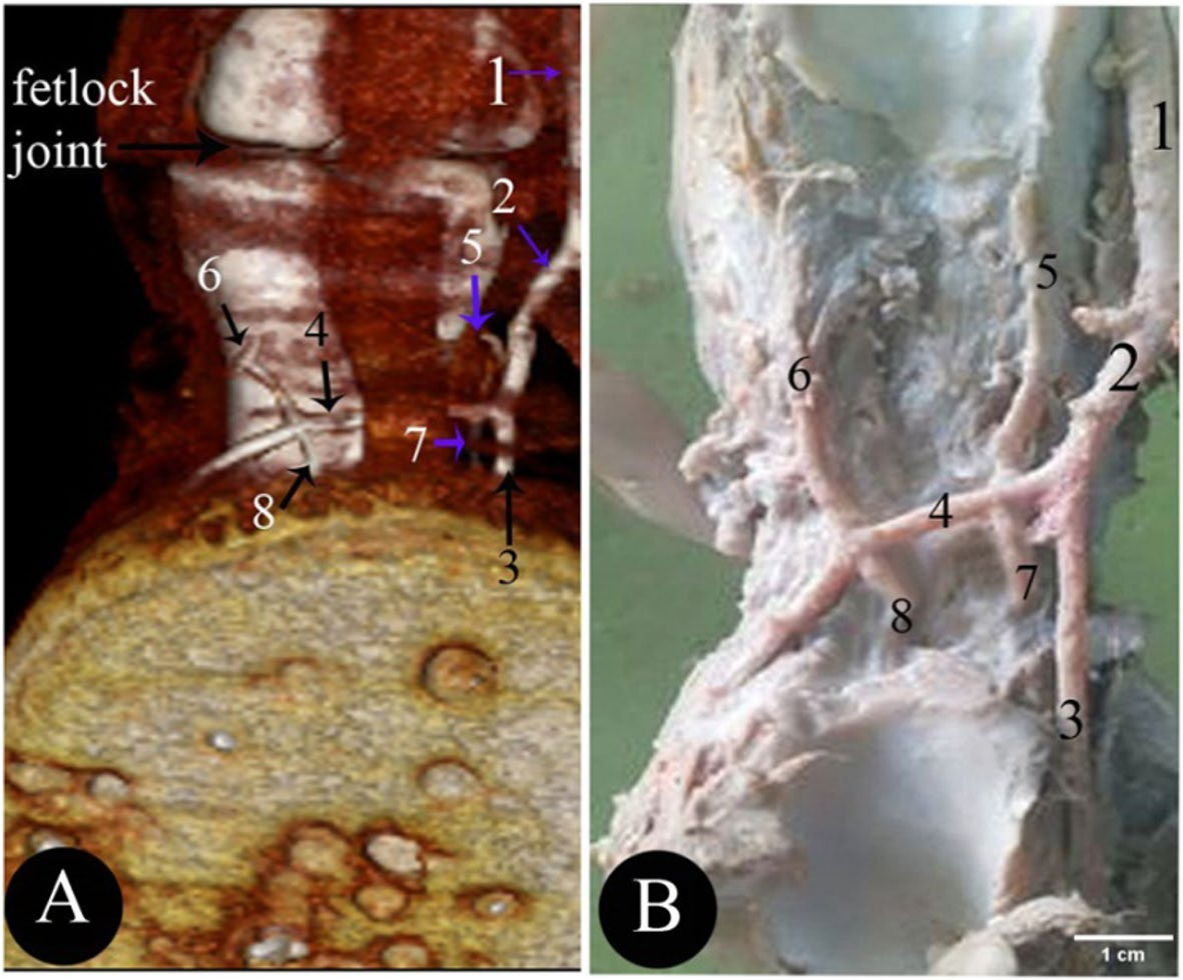
Plantar views of the 3D render volume of the CT (**A**), and gross (**B**) at the level of the 1st phalanx of the left limb of 13 years male camel. 1. *A. digitalis* plantaris communis III, 2. Ramus lateralis of the *A. digitalis* plantaris communis III, 3. *A. digitalis* plantaris propriae III et IV axialis, 4. *A. digitalis* plantaris propriae III et IV abaxialis, 5. Ramus plantaris phalangis axialis proximalis of the first phalanx of the fourth digit, 6. Ramus plantaris phalangis abaxialis proximalis of the first phalanx of the fourth digit, 7. Ramus plantaris phalangis axialis distalis of the first phalanx of the fourth digit, 8. Ramus plantaris phalangis abaxialis distalis of the first phalanx of the fourth digit

The blood supply of the camel hind foot originated mainly from the *A. digitalis plantaris communis III* (Figs. 2/4; 3/1; 4/3; 5/1; 6/1; 7/1; 8/1) which was the direct extension of the *A. plantaris medialis superficialis* at the distal third of the metatarsal (Fig. 2/23). The *A. plantaris medialis superficialis* passed between the digital flexor tendon and the middle interosseus muscle (suspensory ligament) on the abaxial border of the third metatarsal bone (Figs. 2/1, 21, 26; 4/1; 8/12). It deviated diagonally distomedially towards the plantar side of the distal third of the metatarsal bone, before giving out two branches; the *A. digitalis plantaris communis II* and *IV* (Figs. 2/2, 3; 4/2). The *A. digitalis plantaris communis III* (Fig. 2/4) continued distally at the interdigital space between the deep digital flexor tendon and superficial digital flexor tendon (Fig. 4/27, 28; 5/17,18), it gave off a *ramus anastomoticus* cum that united with the *A. metatarsea plantaris III* (Fig. 5/3). The *A. interdigitalis* has arisen from this communication and the *A. digitalis plantaris communis III* in one specimen (Figs. 2/7; 3/5; 4/5; 5/6; 6/3). It gave a *ramus articularis medialis* to the fetlock joint at a level of the proximal half of the first phalanx (Figs. 2/16; 3/7; 5/8), and another *ramus articularis lateralis* to the fetlock joint has arisen from the *ramus lateralis* of the *A. digitalis plantaris communis III* (Figs. 2/17; 5/7). In one specimen, the *ramus articularis lateralis* originated from the *A. digitalis plantaris communis III* itself (Fig. 3/6). Which was divided into *ramus medialis* and *ramus lateralis* (Figs. 2/9,10; 3/9,10, 4/38; 6/2), which passed parallel to the axial border of the 3rd and 4th digit (Figs. 2/9,10; 8/7,8). The *ramus medialis* supplied the third digit (Fig. 6/2), while the *ramus lateralis* supplied the fourth digit. At the half of the first phalanx, the *ramus medialis* and *lateralis* detached the *A. digitalis plantaris propriae axialis and abaxialis* (Figs. 2/11,12; 3/11,12; 4/9,10; 6/2; 8/10,11). In one specimen, the *ramus medialis* of the *A. digitalis plantaris communis III* gave an additional articular branch to the fetlock joint (Fig. 3/8). Additionally, the *A. digitalis plantaris communis III* gave the *ramus superfecialis* (Fig. 4/6).

**Fig. 7:**
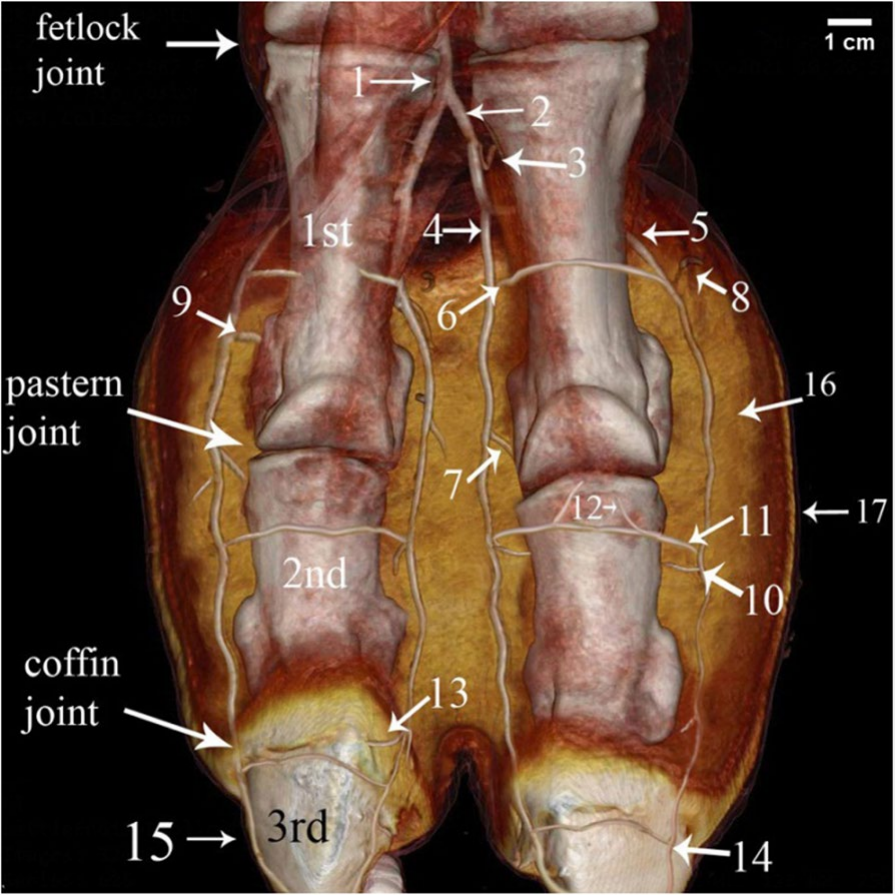
Dorsal view of the 3D render volume of the CT of the bones of the phalanges and the arteries of the left hind digit region of 11 years male camel. 1. *A. digitalis* plantaris communis III, 2. Ramus medialis of the *A. digitalis* plantaris communis III, 3. Ramus articularis of the fetlock joint of the Ramus medialis, 4. *A. digitalis* plantaris propriae III et IV axialis, 5. *A. digitalis* plantaris propriae III et IV abaxialis, 6. Ramus dorsalis phalangis axialis of the first phalanx, 7. Axial Ramus articularis of the pastern joint, 8. Ramus tori digitalis abaxialis, 9. Ramus plantaris phalangis abaxialis of the 1st phalanx, 10. Ramus plantaris phalangis abaxialis of the second phalanx, 11. Ramus dorsalis phalangis abaxialis of the second phalanx, 12. Ramus dorsalis proximalis of the 2nd phalanx, 13. Ramus articularis axialis of the coffin joint, 14. Ramus coronalis of the *A. digitalis* plantaris propriae abaxialis, 15. *A. digitalis* plantaris propriae abaxialis of the third phalanx, 16. Digital cushion, 17. Skin, 1st. First phalanx, 2nd. Second phalanx, 3rd. Third phalanx

**Fig. 8:**
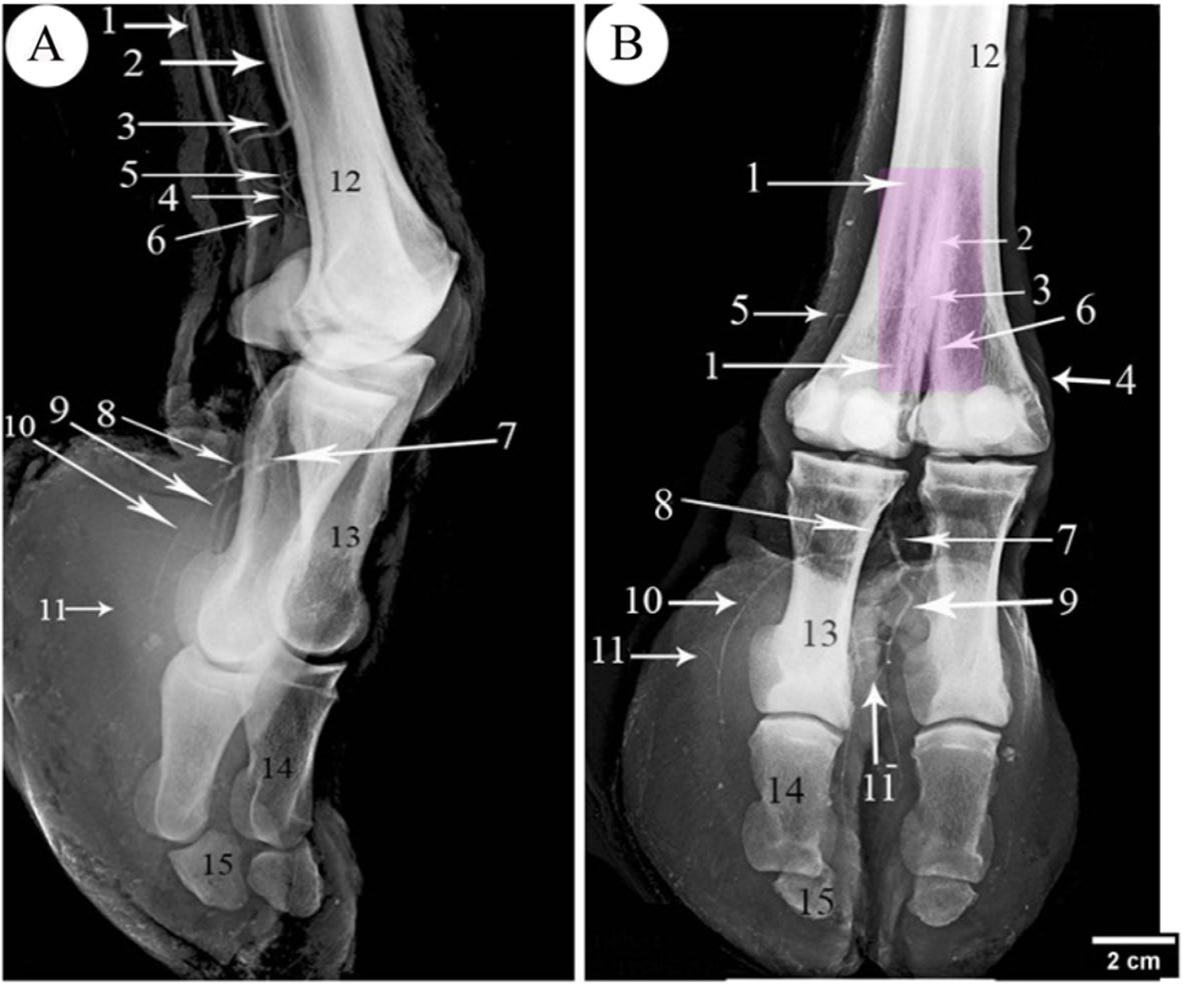
Angiogram showing the arterial pattern of 9 years she-camel right hind foot (**A**), mediolateral, and (**B**) dorsoplantar views. 1. *A. digitalis* plantaris communis III, 2. A. metatarsae plantaris III, 3. Ramus anastomoticus cum between 1& 2, 4. A. metatarsae plantaris II, 5. Metatarsae plantaris IV, 6. Ramus interdigitalis, 7. Ramus lateralis of the *A. digitalis* plantaris communis III, 8. Ramus medialis of the *A. digitalis* plantaris communis III, 9. *A. digitalis* plantaris propriae III et IV axialis, 10. *A. digitalis* plantaris propriae III et IV abaxialis, 11. Ramus tori digitalis abaxialis, 11^−^. Ramus tori digitalis axialis, 12. Third metatarsal bone, 13. First phalanx, 14. Second phalanx, 15. Third phalanx

### *A. digitalis* plantaris propriae III et IV abaxialis

These arteries arose from the *ramus medialis* and *lateralis* of the *A. digitalis plantaris communis III* (Figs. 2/11; 4/9; 5/14,15; 6/4). The *A. digitalis plantaris propriae III et IV abaxialis* passed lateral to the oblique sesamoidean ligament between the first phalanx and the digital flexor tendons before reaching the abaxial aspect of the 3rd and 4th digits (Figs. 8/10). It then continued distally parallel to the abaxial border of each digit till it reached the third digit communicated the axial one to form the *A. coronalis* and *arcus terminalis* (Figs. 2/22; 4/19,20). At the plantar surface of the first phalanx, it gave the *ramus plantaris phalangis abaxialis proximalis and distalis* (Fig. 5/9, 9^−^,10). At the level of the second phalanx, it gave the *ramus dorsalis and plantaris abaxialis* and *ramus articularis abaxialis*. At the level of the third phalanx, it gave the *ramus dorsalis and plantaris abaxialis,* the *ramus coronalis*, and several *rami tori digitalis abaxialis* to the digital cushion (Fig. 8/11).

### *A. digitalis* plantaris propriae III et IV axialis

It was the second branch detached from the *ramus medialis* and *lateralis* of the *A. digitalis plantaris communis III*. It passed through the interdigital space and branched the *ramus dorsalis axialis* of the first phalanx, the *ramus dorsalis axialis* of the second phalanx, *ramus articularis axialis*, *ramus dorsalis axialis* of the third phalanx, the *ramus coronalis*, and the *rami tori digitalis axialis* (Figs. 5/13; 6/3; 8/11^−^).

### Ramus phalangis axialis and abaxialis of the first phalanx

The *ramus plantaris phalangis abaxialis proximalis* of the first phalanx was one artery that ran proximally lateral to the sesamoid ligament at the plantarolateral surface of the first phalanx (Figs. 2/13; 3/13; 4/12; 5/9,9^−^; 6/6). In one specimen, it was anastomosed with the *ramus articularis* of the medial one and perforated the fetlock joint capsule (Fig. 4/8). There was also a *ramus axialis* in one specimen (Fig. 6/5).

The *ramus plantaris phalangis abaxialis distalis* of the first phalanx was observed in one artery on the opposite side of the proximal one (Figs. 2/14; 4/11; 5/10; 6/8). There was also a *ramus axialis* in one specimen (Fig. 6/7).

The *ramus plantaris phalangis axialis and abaxialis* of the first phalanx were detached from the main proper digital branch corresponding to the *ramus dorsalis*. It ranged from one to two branches, it presented mainly at the distal third of the first phalanges, and it crossed the plantar surface of the first phalanx and sesamoidean ligament to anastomoses with the corresponding side (Figs. 2/15; 4/16; 5/11; 7/9; 8/9).

The *ramus dorsalis phalangis axialis and abaxialis* of the first phalanx were one or two fine arteries that arose at the middle half of the first phalanx (Figs. 4/13; 5/15). They passed to the dorsal aspect of the first phalanx under the common digital extensor tendon and the two arteries anastomosed (Fig. 7/6).

The *rami articularis axialis and abaxialis* of the pastern joint were about one or two fine branches at each side that entered the pastern joint (Figs. 2/18; 4/15; 5/12; 7/7).

### Ramus phalangis axialis and abaxialis of the second and third phalanges

The *ramus dorsalis phalangis axialis and abaxialis* of the second phalanx was one branch emerged from each side of the second phalanx and anastomosed at the middle (Figs. 4/17; 7/11). It passed nearly the proximal third of the second phalanx and gave a small *ramus dorsalis proximalis* to the pastern joint (Fig. 7/12).

The *ramus plantaris phalangis axialis and abaxialis* of the second phalanx were given separately or by a common stem with the dorsal one, they passed nearly at the proximal half of the second phalanx. They supply the plantar surface of the corresponding bone, ramified to the digital cushion, and finished in the plantar surface of the second phalanx (Figs. 2/19; 7/10).

The *rami articularis axialis and abaxialis* of the coffin joint were observed with one fine branch at each side passing toward the coffin joint (Figs. 2/20; 4/18; 7/13).

The *ramus dorsalis phalangis axialis and abaxialis* of the third phalanx emerged from the *A. digitalis plantaris propriae axialis and abaxialis* (Figs. 2/21, 22; 4/20; 7/15). The anastomoses with each other formed a terminal arterial plexus *(arcus terminalis)*, which embraced the third phalanx (Fig. 2/27).

The *rami tori digitalis axialis and abaxialis* were 7–10 branches on each side, which detached from the *A. digitalis plantaris propriae axialis and abaxialis* along the three phalanges (Figs. 4/14; 7/8). They ran in a distal direction and entered the digital cushion, where they detached fine branches to the sole and digital cushion (Figs. 4/33; 7/16,17; 8/11,11^−^).

The *ramus coronalis* detached from the *A. digitalis plantaris propriae abaxialis* (Figs. 4/19; 7/14), it passed dorsally, then axially near the coronary border of the third phalanx, and it anastomosed with the corresponding axial one to form the *arcus coronalis* just dorsal to the coffin joint.

### *A. Metatarsae plantaris III* (Figs. 2/5; 3/2; 4/4; 5/2; 7/2; 8/2)

It passed at the dorsal longitudinal groove between III and IV metatarsus bone (Figs. 2/23,24; 4/21,22) and was covered by the middle interosseous muscle (Fig. 4/26). A *ramus communes* was observed between this artery and the *A. digitalis plantaris communis III* (Figs. 5/3; 7/3; 8/3) that gave three branches; *ramus interdigitalis* (Figs. 3/5; 4/5; 8/6), *A. metatarsae plantaris II and IV* (Figs. 2/6; 2/8; 3/4; 4/7,8; 5/4,5; 7/4,5; 8/4,5) under the branches of the suspensory ligament (middle interosseus muscle) (Fig. 3/3; 5/16) where they ramified around the medial and lateral condyles of the third and fourth metatarsal bones (Fig. 8/12) and gave many branches, the main one was the *ramus articularis* that was directed to the fetlock joint (Figs. 4/4; 5/5^−^). The *Ramus articularis* to the fetlock joint of the A. metatarsae plantaris III passed under the abaxial collateral ligament of the fetlock joint and dorsal to the abaxial collateral sesamoidean ligament until it reached the joint cavity (Fig. 5/19, 20). The ramus interdigitalis emerged from the *A. metatarsae plantaris III* in all studied camels except in one camel where it emerged from the *A. digitalis plantaris communis III* (Fig. 2/7).

Finally, all the main arterial variations at the plantar aspect of the distal part of the metatarsus and digit of the studied camel are demonstrated in Table 2.

**Table 2:** The main arterial variations at the plantar aspect of the distal part of the metatarsus and digit in camel

The artery	Commonly	Variability
• Ramus interdigitalis	• It arose from the ramus anastomoticus cum between the *A. digitalis* plantaris communis III and the A. metatarsae plantaris III (Figs. 3/5; 4/5; 8/6).	• It arose from *A. digitalis* plantaris communis III in one case (Fig. 2/7).
• Ramus articularis to the fetlock	• The ramus articularis medialis has arisen from the *A. digitalis* plantaris communis II (Figs. 2/16; 3/7; 5/8). • The ramus articularis lateralis has arisen from the Ramus lateralis of the *A. digitalis* plantaris communis III (Fig. 5/7).	• In one case, the ramus articularis lateralis originated from the *A. digitalis* plantaris communis II itself (Fig. 3/6).
• Ramus plantaris phalangis abaxialis proximalis of the 1st phalanx	• Only one abaxial branch (Figs. 2/13; 5/9).	• In one case, there was also an axial branch (Fig. 6/5). • In one case, it anastomosed with the articular branch of the lateral one and perforated the fetlock joint capsule (Fig. 5/7,8).
• Ramus plantaris phalangis abaxialis distalis of the 1st phalanx	• Only one abaxial branch (Figs. 2/14; 4/11).	• In one case, there was also an axial branch (Fig.6/7).

## Discussion

Camels generally belong to two species, the double-humped Bactrian camel and one-humped camel (dromedary camel). They are located in African and Asian deserts where they adapt to high temperature, water and food scarcity for longer periods. It is known as the ship of the desert and can tolerate water losses greater than 25% of its weight [31, 32]. The smaller-size camels in South America are the domesticated llama. The llama is related to camels but without the hump. The camel and llama were used for transportation and trading as a source of meat, milk, and wool [33, 34]. The camel and llama possessed some anatomical and physiological similarities as a similar number of diploid chromosomes, and the left horn is larger than the right one [34–36]. Llama has a small size, cloven hoof, and dense wool layer until tolerating a low temperature, while the camel had one broad footpad and scaly hair coat [37].

The 3D render volume reconstruction of the digit regions gave us a better visualization of the bones of the digits from all sides, in addition to slight flexion of the fetlock joint, which gave us a chance to explore the articular surfaces of the different parts of the digit bones. 3D reformatted images permit a better approach and understanding of temporal bone anatomy and possess the capability to value related diseases therby enabling radiologists to spot and demonstrate individual osseous and soft-tissue components of complex anatomy [38, 39]. The 3D reconstruction of the bones demonstrated all the structures of the digit bones and explained the bones of the lateral and medial sides, the right and left limbs of the same leg were nearly similar in size. The current study indicated that from the measurements of the phalanges, the left side had a slight increase in values over the right side while the lateral sides had higher values than the medial sides [5–7].

Computed tomography images are helpful for veterinary students for a better description of the anatomical structure. The 3D computed tomography delivered a better demonstration for the anatomical CT and can produce virtual casts. The 3D image can be printed on a 3d printer and give the anatomical models to facilitate the learning and better understanding and benefit to research and diagnoses process [23, 40].

Angiography X-rays and CT produce detailed images of the arteries and tissues of the foot. Angiography has many problems mainly one is superimposition, two-dimension the relation with other structures is difficult except with the bones. The CT modality has evolved in recent years because of its high-resolution 3D data [41]. Contrast-enhanced CT of vascular imaging is extremely desirable, but the attenuation of blood vessels is usually too low to detect directly via CT imaging, to beat its limitation, vascular structures might be visualized by 3D CT analysis combined with a perfused contrast agent [42]. Multi-slice Computed tomography angiography gives fast scan and good resolution and the ability to render volume three-dimensional (3D) images [43].

The present study documented that the main blood supply of the Dromedary Camel digit was the *A. digitalis plantaris communis III* and the *A. metatarsae plantaris III*. While [12, 13] in the Dromedary Camel and llama denoted that the *A. digitalis plantaris communis III* was the main blood supply of the hind limb digit. However, the blood supply of the camel forelimb digit was derived from the *A. metacarpae plamaris III* and the *A. digitalis plamaris communis III*.

Our study explained a *ramus anastomoticus* cum between the *A. metatarsae plantaris III* and the *A. digitalis plantaris communis III* that gave three branches; *ramus interdigitalis*, *A. metatarsae plantaris II,* and *IV.* While [2] in camel stated that *A. metatarsae plantaris II* and *IV* come from the *A. metatarsae plantaris III*. In llama the *A. plantaris* medialis superficialis reaches the hind foot, it might be named the *A. digitalis plantaris communis III* [13]. In addition, the anastomosis between the *A. digitalis plantaris communis III* and the *A. metatarsae plantaris III* produces the *A. digitalis plantaris communis II* and *IV.*

The current study recorded variable sources of the *ramus interdigitalis*, which arose from the *A. digitalis plantaris communis III*, or the *ramus anastomoticus* cum between the *A. metatarsae plantaris III* and the *A. digitalis plantaris communis III*. Despite [2] in the Dromedary Camel mentioned that the source of the *ramus interdigitalis* was the *A. metatarsae plantaris III*.

In our work, we recorded that only one abaxial branch was detached from the branches of the *rami plantaris phalangis abaxialis proximalis* and *distalis* of the first phalanx, there were *rami plantaris phalangis axialis proximalis* and *distalis* recorded in one case. While [12, 13] in the Dromedary Camel and llama, mentioned that the *rami plantaris phalangis abaxialis proximalis* and *distalis* of the first phalanx were absent. The abaxial branch was noticed in the Dromedary Camel forelimb [20].

We noticed the *ramus plantaris phalangis abaxialis proximalis* of the first phalanx share with the *ramus* articularis from the *A. digitalis plantaris communis III* or from its two branches to supply the fetlock joint. In addition, the *ramus* articularis from the *A. metatarsae plantaris II* and *IV* also supplied the fetlock joint. While [2] in the Dromedary Camel stated that only the *A. metatarsae plantaris II* and *IV* were the sources of the fetlock blood supply. There was not a relationship between the variation of the arteries and the age and sex of the Dromedary Camel.

Our study explained that the *rami tori digitalis axialis* and *abaxialis* were 7–10 branches on each side to supply the hind limb digital cushion whereas the adaptation of the camel to desert conditions requires more blood supply. While [14] in Bactrian camel reported 6–7 axial and 7–9 abaxial *rami tori digitalis*. The *rami tori digitalis* was less massive in the ox and buffalo. In the Dromedary Camel, the slight increase of the hind limb digital tori branches than at the forelimb may be due to the hind limb having high load than fore one. We can detect the relation of arteries with ligaments as the *A. plantaris medialis superficialis* pass between the digital flexor tendon and the suspensory ligament. The *A. digitalis plantaris propriae III et IV abaxialis* passed between the lateral to sesamoid ligament and the digital flexor tendons. The *A. metatarsae plantaris II* and *IV* were demonstrated under the branches of the suspensory ligament this like that was recorded by [4].

The digital circulation angiography explores the circulatory pattern of the camel hind digit that can help the clinicians through the diagnosis of the digit region affections. Many affections of the digit can be identified such as thrombosis of the digit sole, altered circulation into the dermis of the sole, largely undeveloped terminal arterial arches, small or undeveloped primary arterial arches, and small or irregular arterial extension to the corium of the coronary band [44]. The common causes of camel lameness were reported as a musculoskeletal disorder at phalangeal and foot disorders [45].

Amputation of the digit at any level requires a knowledge and understanding of the digital anatomy and its blood supply is important before performing surgeries [46]. The amputation level in camels varied according to the severity; the surgical incision may be at a level between the distal phalanx and nail or the pastern and the fetlock joints [47].

Because, despite the superimposition, these results were matched with [48], 3D CT to bone and arteries provides better visualization and relationship of the structures to each other than normal CT and angiography. Volume rendering (VR) is today’s standard three-dimensional (3-D) image for visualizing complex anatomical information.

## Data Availability

The datasets used and/or analyzed during the current study are available from the corresponding author on reasonable request.
